# Ipsilateral S2 nerve root transfer to pudendal nerve for restoration of external anal and urethral sphincter function: an anatomical study

**DOI:** 10.1038/s41598-019-50484-7

**Published:** 2019-09-30

**Authors:** Lei Zhu, Zhi-bin Zhou, Di Shen, Ai-min Chen

**Affiliations:** Department of Orthopedic Trauma Surgery, Changzheng Hospital, Second Military Medical University, Shanghai, China 200003

**Keywords:** Peripheral nervous system, Somatic system

## Abstract

Patients suffer bilateral sacral plexus injuries experience severe problems with incontinence. We performed a cadaveric study to explore the anatomical feasibility of transferring ipsilateral S2 nerve root combined with a sural nerve graft to pudendal nerve for restoration of external anal and urethral sphincter function. The sacral nerve roots and pudendal nerve roots on the right side were exposed in 10 cadavers. The length from S2 nerve root origin to pudendal nerve at inferior border of piriformis was measured. The sural nerve was used as nerve graft. The diameters and nerve cross-sectional areas of S2 nerve root, pudendal nerve and sural nerve were measured and calculated, so as the number of myelinated axons of three nerves on each cadaver specimen. The length from S2 nerve root to pudendal nerve was 10.69 ± 1.67 cm. The cross-sectional areas of the three nerves were 8.57 ± 3.03 mm^2^ for S2, 7.02 ± 2.04 mm^2^ for pudendal nerve and 6.33 ± 1.61 mm^2^ for sural nerve. The pudendal nerve contained approximately the same number of axons (5708 ± 1143) as the sural nerve (5607 ± 1305), which was a bit less than that of the S2 nerve root (6005 ± 1479). The S2 nerve root in combination with a sural nerve graft is surgically feasible to transfer to the pudendal nerve for return of external urethral and anal sphincter function, and may be suitable for clinical application in patients suffering from incontinence following sacral plexus injuries.

## Introduction

Sacral plexus injury caused by high-energy traumas can lead to severe disabilities including motor dysfunction in the lower extremities, as well as significantly impaired bowel, bladder, and sexual function^1,2^. The complexity of these injuries complicates functional reconstruction of sacral plexus injury by limiting surgical options, thus few treatment strategies are available for clinical application, resulting in poor and insufficient functional recovery. In recent years, some surgical methods such as direct anastomosis^3^ and nerve transfer^4^ have been developed, which achieved certain functional recoveries in lower extremities. In adition, patients with sacral fractures who suffer bilateral sacral plexus injuries also experience severe problems with incontinence^5,6^. Due to the denervation of external anal and urethral sphincter after sacral nerve injury, the incontinence problem eventually arises. However, relatively few studies concerning incontinence have been reported, despite worser functional outcomes.

Nerve transfer is a well-recognised surgical procedure for the restoration of nerve function when direct anastomosis was unable to be conducted after injury^7,8^. Many patients with sacral fractures who suffer from incontinence show intra-operative findings of stretched, contused, or lacerated sacral nerve roots, so that the distal nerve can hardly been indefied in the sacral canal. As a result, functional recovery was impossible to be achieved through decompression of fracture, direct anastomosis or nerve grafting of sacral nerve roots. In many cases, except for the most severe sacral nerve roots avulsion, the nerve roots at their origin were commonly intact, making it possible to repair the function of distal nerve by combining proximal nerve root with sural nerve transplantation. Therefore, with an attempt to restoring continence, we hypothesized that the S2 nerve in combination with a sural nerve graft could be transferred to pudendal nerve for restoration of external urethral and anal sphincter function. However, the anatomical feasibility of the novel procedure needs to be tested before its clinical application. As a result, we reported the results of a cadaveric study performed to assess the feasibility.

## Materials and Methods

**Figure 1:**
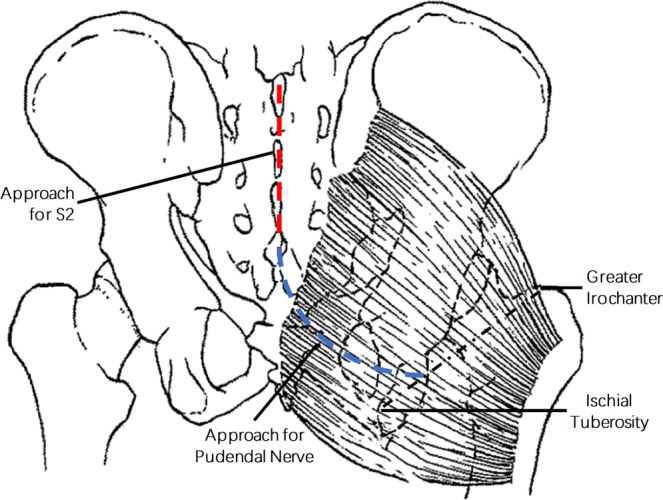
Diagram illuminating approachs for exposure of S2 nerve root and pudendal nerve.

**Figure 2:**
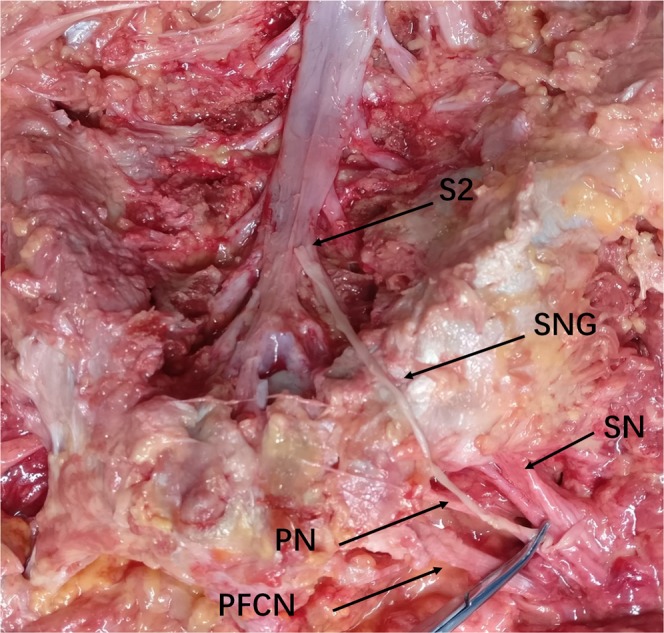
Cadaveric picture showing the transfer of right S2 nerve root to the right pudendal nerve. S2, S2 nerve root; PN, pudendal nerve; SN, sciatic nerve; SNG, sural nerve graft; PFCN, posterior femoral cutaneous nerve.

Ten fresh adult cadavers (5 male and 5 female) were used in this study. The cadavers were obtained from the Anatomy Department of Second Military Medical University. The cadavers were dissected prone on the operation table. The spinal cord and nerve roots were exposed through a posterior midline approach and a standard posterior sacral laminectomy (Fig. 1). As for approach to expose pudendal nerve, a curved incision was made from the end of sacral incision to the interior 1/3 point of the junction of the greater trochanter of femur and the ischial tuberosity (Fig. 1). After dissection of skin and subcutaneous tissue, exposure of piriformis were made by blunt dissection of gluteus maximus along its muscle fibers. Then, the pudendal nerve, sciatic nerve, posterior femoral cutaneous nerve were identified at the inferior border of piriformis. The right sural nerve was also dissected for nerve graft through approach as previous reported^9^. In order to obtain maximal nerve roots and pudendal nerve exposure for measurement, the right gluteus maximus and all the tissues between approachs for nerve roots and pudendal nerve exposure were removed on cadavers (Fig. 2). After the nerve dissection was completed, the distance from the right S2 nerve root origin to the pudendal nerve at inferior border of piriformis was measured by a flexible measuring tape. Then, a sural nerve of which the length is five centimeters longer than the measured distance was dissected and used for tension-free neurorrhaphy. The transverse diameter (TD) and longitudinal diameter (LD) of the pudendal nerve, S2 nerve root and pudendal nerve were measured with a caliper, and the cross-sectional areas (CA) of nerves were calculated according to the formula: CA = TD × LD × **π**(3.14159)/4. We also obtained 1-cm segments of the pudendal nerve, S2 nerve root and pudendal nerve for pathological section and toluidine blue staining. The sections of the nerves were fixed in formaldehyde overnight, serially dehydrated in alcohol and embedded in Epon. Cross sections (thickness, 0.5μm) were cut and stained with 1% toluidine blue, examined under a 400× microscope (Leica, Wetzlar, Germany). The number of myelinated axons of each nerve was then calculated byma Leica FW4000 image analysis system (Leica, Wetzlar, Germany). All quantitative data are presented as the mean ± SD.

The research was in compliance with the Helsinki Declaration. All experimental protocols were approved by the Ethics Committee of the Second Military Medical University (Shanghai, China), and informed consent for the use of the cadavers in the study has been obtained before experiment.

## Results

**Figure 3:**
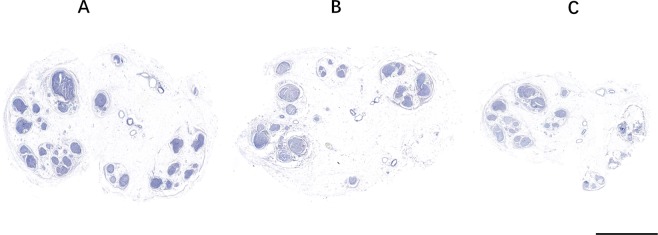
Representative neuromorphometric images of nerve cross sections. A, S2 nerve. B, pudendal nerve. C, sural nerve. Scale bar = 1 mm.

The results of anatomical measurements are presented in Table 1.The average length from S2 nerve root to pudendal nerve was 10.69 ± 1.67 cm, and a sural nerve graft which was five centimeters longer than this distance was adequate for tension-free neurorrhaphy between S2 nerve root and pudendal nerve, as indicated in Fig. 2. The transverse diameter and and longitudinal diameter of S2 nerve root were 2.98 ± 0.57 mm and 3.55 ± 0.71 mm, while the diameters of sural nerve and pudendal nerve were almost the same (2.77 ± 0.44 mm vs. 2.62 ± 0.39 mm in TD and 3.16 ± 0.46 mm vs. 3.03 ± 0.38 mm in LD), as showed in Table 1. After calculating, the results showed that cross-sectional areas of the three nerves were similar (8.57 ± 3.03 mm^2^ for S2, 7.02 ± 2.04 mm^2^ for pudendal nerve and 6.33 ± 1.61 mm^2^ for sural nerve). Thus, nerve anastomosis of the S2 root and the pudendal nerve to the sural nerve without tension was possible. Furthermore, the pudendal nerve contained approximately the same number of axons (5708 + 1143) as the sural nerve (5607 + 1305), which was a bit less than that of the S2 nerve root (6005 + 1479), demonstating that both the S2 root and sural nerve have a sufficient number of motor axons to connect with pudendal nerve and power its innervated muscles (Fig. 3).

**Table 1:** Measurements (Mean ± SD) of the nerves on cadavers.

	TD(mm)	LD(mm)	CA(mm^2^)	N	L(cm)
S2	2.98 ± 0.57	3.55 ± 0.71	8.57 ± 3.03	6005 ± 1479	
PN	2.77 ± 0.44	3.16 ± 0.46	7.02 ± 2.04	5708 ± 1143	
SN	2.62 ± 0.39	3.03 ± 0.38	6.33 ± 1.61	5607 ± 1305	10.69 ± 1.67

Note: Values are presented at the Mean ± SD. S2, S2 nerve root; PN, pudendal nerve; SN, sural nerve; TD, transverse diameter; LD, longitudinal diameter; CA, cross-sectional area; N, number of myelinated axons; L, distance from the right S2 nerve root origin to the pudendal nerve at inferior border of piriformis.

## Discussion

Pelvic and sacral fractures caused by high-energy trauma are rare but serious injuries, with which neurologic disorder is frequently associated. Sacral fractures involving the sacral plexus, especially bilateral sacral plexus, have a high prevalence of neurological injury, leading to severe disabilities including impaired motor function, loss of sensation in the lower extremities, as well as bowel, bladder, and sexual dysfunction^10^. Although the incidence of neurological injury is high, the functional recovery remains uncertain^11^. As a result of the poor functional outcomes after surgery in cases of transection, compression, and avulsion of sacral nerve roots^12–16^, conservative treatment has been advocated.

In recent years, functional recovery in lower-extremities after surgery in cases of lumbosacral plexus injuries has been reported. Many surgeons^2,5,15^ reported patients with compressed but intact nerve roots caused by sacral fracture that treated with decompression surgery to achieve neurological recovery. Denis^12^ *et al*. reported neurological improvement in five patients following decompression and encouraged early decompression. Schmidek^17^ *et al*. found bowel and bladder dysfunction improvement in patients with transverse sacral fractures after surgical decompression. Zelle^2^ *et al*. proved that most of sacral fractures associated with neurological injuries benefit from surgical decompression in a retrospective study.

In addition to the compressed but intact nerve roots following sacral fractures, many intra-operative findings have also shown stretched, contused, or lacerated nerve roots, in which of the situations the distal nerve can hardly been indefied in the sacral canal. As a result, functional recovery was impossible to be achieved through decompression of fracture, direct anastomosis or nerve grafting of sacral nerve root^1,4,5^. Nerve transfer is a validated surgical procedure for the restoration of nerve function after severe nerve injuries and the keypoint of the surgery is to find effective and convenient donor nerves. Brunelli^18^ *et al*. transferred the ulnar nerve to repair the femoral, superior gluteal, and obturator nerves. Thomas^19^ *et al*. described successful restoration of quadriceps function by obturator nerve transfer in two patients. Garcia-Lopez^20^ *et al*. transferred the brachioradialis motor branch to the anterior interosseous nerve in an anatomic and histomorphometric study. Previous cadaveric studies also examined the use of nerve transfer to restore bowel and bladder functions in dogs and cadavers^21–26^. In addition, studies showed that spinal roots could also be used as donor nerves for functional reconstruction of neural injuries. For example, C7 and S1 nerve root transfer from the contralateral side have been used successfully for the treatment of brachial and sacral plexus avulsion, which were considered impossible to repair before^4,8^. Although various nerve transfer procedures have been designed to improve upper and lower extremities, bowel and bladder functions following nerve injuries, the use of sacral nerve root as donor nerve to repair external urethral and anal sphincter function has not been reported.

Patients with transverse sacral fracture frequently suffer from bowel and bladder incontinence which damages their life qualities. It will be a great improvement with the restoration of bowel and bladder control. While Ruggieri^26^ *et al*. reported a canine study of femoral nerve to pudendal nerve transfer to restore urethral and anal sphincter function, most pevious studies focused on restoration of bladder emptying but few on restoration of sphincter control^21–25^. In the present study, we tried to restore continence by reconstructing anal and urethral sphincter function via nerve root transfer. Since the anal and urethral sphincter are innervated by the pudendal nerves, and the S2 root is one of the origin of the pudendal nerve, the anatomical feasibility of transferring the S2 nerve root to the ipsilateral pudendal nerve for the restoration of anal and urethral sphincter function was examined. Although S2 nerve root was commonly intact at its origin following sacral fractures, direct anastomosis of the S2 nerve root and pudendal nerve was impossible to be achieved because of large nerve gap from S2 to pudendal nerve in the pelvis. As a result, the use of an nerve graft is essential. In our study, sural nerve was chosen as nerve graft, which has been widely used in the repair of sciatic nerve, femoral nerve, common peroneal nerve and even axillary nerve^27–30^ with mild residual symptoms of doner nerve^31^. We found that the sural nerve root contained approximately the same number of axons as the pudendal nerve, although less than that of the S2 nerve root. The number of axons in the S2 nerve root could suffice for pudendal nerve regeneration. We measured the distance between S2 nerve root origin to pudendal nerve at inferior border of piriformis as a reference for the length of sural nerve graft. Our data showed that a sural nerve graft which was five centimeters longer than this distance was adequate for tension-free neurorrhaphy between S2 nerve root and pudendal nerve. According to previous study, the average length of sural nerve for adult is about 18.3 cm^9^, indicating sural nerve is an ideal option for autografting and sufficient in length to provide a tensionless repair. What’s more, the diameters and cross-sectional areas of S2 nerve root, sural nerve and pudendal nerve were similar, which also illuminated that nerve anastomosis of the S2 root and the pudendal nerve to the sural nerve without tension was possible. Therefore, we believe that the S2 nerve root in combination with a sural nerve graft is feasible for surgical transfer to repair pudendal nerve following sacral plexus injury.

The major limitation of this study is similar to those of previous studies. This is a cadaveric study and further experiments on animals are still needed before clinical application. However, our study presents the anatomical feasibility of S2 nerve root transfer to pudendal nerve. The exposure and anastomosis of nerves were relatively easy, which has great potential in clinical applications of patients with loss of pudendal nerve function. Additionally, although we presented a feasible surgery to restore continence in our study, the bladder emptying function would need to be accomplished by other means such as abdominal pressure or clean intermittent catheterization.

## Conclusion

The results of this anatomical study revealed that a S2 nerve root in combination with a sural nerve graft could be transferred to pudendal nerve for the repair of external urethral and anal sphincter function in patients suffering from incontinence following sacral plexus injuries.
